# ALYREF promotes the metastasis of nasopharyngeal carcinoma by increasing the stability of NOTCH1 mRNA

**DOI:** 10.1038/s41419-024-06959-1

**Published:** 2024-08-08

**Authors:** Yanan Jin, Jijin Yao, Jianchang Fu, Qitao Huang, Yilin Luo, Yafei You, Wangjian Zhang, Qian Zhong, Tianliang Xia, Liangping Xia

**Affiliations:** 1 VIP Region, State Key Laboratory of Oncology in South China, Guangdong Key Laboratory of Nasopharyngeal Carcinoma Diagnosis and Therapy, Sun Yat-sen University Cancer Center, Guangzhou, Guangdong PR China; 2 The Cancer Center of the Fifth Affiliated Hospital of Sun Yat-sen University, Zhuhai, Guangdong PR China; 3 The Cancer Center of Nasopharyngeal Carcinoma, the Fifth Affiliated Hospital of Sun Yat-sen University, Zhuhai, Guangdong PR China; 4 Department of Pathology, Sun Yat-sen University Cancer Center, Guangzhou, Guangdong PR China; 5 State Key Laboratory of Oncology in South China, Guangdong Key Laboratory of Nasopharyngeal Carcinoma Diagnosis and Therapy, Sun Yat-sen University Cancer Center, Guangzhou, PR China; 6 Department of Clinical Oncology, The Seventh Affiliated Hospital, Sun Yat-sen University, Shenzhen, Guangdong PR China; 7 Department of Medical Statistics, School of Public Health, Sun Yat-sen University, Guangzhou, Guangdong PR China

**Keywords:** Cell invasion, Head and neck cancer

## Abstract

Approximately 70% of treatment failures in nasopharyngeal carcinoma (NPC) patients are attributed to distant metastasis, yet the underlying mechanisms remain unclear. RNA 5-methylcytosine (m5C) is an emerging regulatory modification that controls gene expression and plays a critical role in tumor progression. However, there is little information on the potential roles of RNA m5C modification in NPC metastasis. In this study, we found that the m5C reader Aly/REF export factor (ALYREF) is significantly upregulated in NPC, whereby its high expression is associated with metastasis and poor prognosis. ALYREF overexpression was found to promote tumor metastasis of NPC cells in vitro and in vivo. Mechanistically, m5C-modified NOTCH1 mRNA was identified as a target of ALYREF. Moreover, ALYREF was found to upregulate NOTCH1 expression by enhancing its RNA stability in an m5C modification-dependent manner, thereby promoting the activation of the NOTCH signaling pathway and facilitating NPC metastasis. Overall, our data reveal the crucial role of ALYREF in NPC metastasis and provide a potential therapeutic target for NPC.

## Introduction

Nasopharyngeal carcinoma (NPC) is particularly prevalent in Southern China, Southeast Asia, and North Africa, with high rates of invasion and metastasis [1]. With advances in NPC treatment, the five-year local control rate for this disease has reached up to 95% [2]. However, 20-30% of patients still experience distant metastases after radical chemoradiotherapy [2, 3]. In spite of recent advances, the prognosis remains poor once distant metastases occur, with a median progression-free survival of 9.6−21.4 months [4, 5]. However, the metastatic mechanism of NPC remains unclear, and understanding the underlying molecular pathways is of great importance for improving the control of distant metastases.

Recently, epigenetic modifications have been confirmed to be important for the initiation and progression of various types of cancer. 5-Methylcytosine (m5C), first reported in the 1970s [6], is a common post-transcriptional modification in eukaryotic mRNAs that plays critical roles in biological processes such as mRNA stability [7, 8], translation efficiency [9], and nuclear export [10]. A series of regulators have been confirmed to be involved in m5C modification of RNAs, including m5C methyltransferases (NSUN1-7, DNMT1-2, and DNMT3A-B) and demethylases (TET2) [11]. Among them, NSUN2 and NSUN6 are two major methyltransferases that catalyze the m5C modification of mammalian mRNAs [10, 12], which exerts its effects by recruiting reader proteins such as Y-box binding protein 1 (YBX1) [8] and Aly/REF export factor (ALYREF) [10]. For instance, YBX1 was found to recruit ELAV-like RNA-binding protein 1 (ELAVL1) and improve the stability of target mRNAs in bladder tumors [8]. In addition, YBX1 was found to bind to AURKA mRNA to enhance its protein level in NPC cells [13]. Previous studies have implied a potential function of ALYREF in facilitating the export of m5C-modified mRNAs [8], as well as its possible role as a positive regulator of tumor growth and metastasis in cancer cell lines [14, 15]. However, the specific roles of ALYREF in NPC remained unclear.

The evolutionarily conserved Notch pathway plays an important role in determining the fate of tumor cells. Among the four NOTCH receptor family members, NOTCH1 has been widely studied due to its high mutation rate in tumors [16]. NOTCH1 has vital functions in important cellular processes such as apoptosis, metastasis, differentiation, and proliferation [17, 18]. In NPC, Hou et al. [19] suggested that overexpression of NOTCH1 enhances cell invasion and migration, but the underlying mechanism remained unclear. Moreover, a recent study demonstrated that activation of the Notch signaling pathway is related to METTL3-mediated m6A (N6-methyladenosine) mRNA modification [20]. However, the roles of other RNA modifications in the Notch signaling pathway remain unclear.

Here, we show that the m5C reader ALYREF is upregulated in NPC tissues and its high expression is associated with a poor prognosis. Moreover, ALYREF interacts with the m5C-modified NOTCH1 mRNA to enhance its stability, thereby activating oncogenic Notch signaling to promote NPC metastasis. Overall, our findings demonstrate that ALYREF is a promising prognostic biomarker and a potential therapeutic target for the treatment of NPC.

## Materials and methods

### Clinical specimens

For expression analysis, we obtained 22 freshly frozen NPC tissues and 10 non-NPC tissues (NPN), which were immediately transferred into RNAlater solution (R0901, Sigma), incubated at 4 °C overnight and then stored at −80 °C. The expression levels of NSUN2 and ALYREF were measured using real-time quantitative polymerase chain reaction (qRT-PCR). In addition, immunohistochemistry (IHC) was performed on 192 NPC tissue biopsies (161 non-metastatic and 31 metastatic NPC tissue samples) and 22 non-NPC tissues, which were independently scored by two pathologists. From June 2010 to May 2015, we collected 161 paraffin-embedded tissue samples from NPC patients (Table S1) for prognostic analysis. Distant metastasis-free survival (DMFS) and overall survival (OS) were considered as endpoints. All patients provided written informed consent. This study was approved by the institutional ethical review board of Sun Yat-sen University Cancer Center (human cancer specimens: SL-B2022-046-01; in vivo mouse experiments: L102012021040B). Principles of the Declaration of Helsinki were followed.

### Cell culture

The authenticated Bmi1-immortalized primary nasopharyngeal epithelial cell lines (NPEC2-Bmi1 and NPEC5-Bmi1) and NPC cells were kindly provided by Professor Mu-Sheng Zeng (Sun Yat-sen University Cancer Center, SYSUCC). NPEC5-Bmi1 and NPEC2-Bmi1 were grown in serum-free keratinocyte medium (Life Technology, USA) with the addition of bovine pituitary extract (BD Biosciences). The NPC cell lines CNE1, CNE2, HONE1, SUNE1, SUNE2, HNE1, S26, S18, and HK1 were cultured in RPMI 1640 medium supplemented with 5% fetal bovine serum (10% FBS for C666), in a humidified incubator with 5% CO_2_ at 37 °C. All cell cultures were tested to ensure that they were free from Mycoplasma infection.

### IHC staining

The IHC procedure was performed according to a previous report [21]. The antibodies and concentrations used were as follows: anti-ALYREF (1:200, HPA019799, Sigma); anti-NSUN2 (1:4000, 20854-1-AP, Proteintech); anti-NOTCH1 (1:100, ab52627, Abcam). Two independent pathologists scored the slides according to staining intensity and area, resolving disagreements through consensus. The immunoreactivity-scoring (IRS) system (a semi-quantified scoring criterion) [22] was used for IHC staining analysis. The staining intensity was designated as follows: no staining (0 points), weak staining (1 point), moderate staining (2 points), and strong staining (3 points). The proportion of cell staining was scored as follows: no staining (0 points), <25% of cells stained (1 point), 25−50% (2 points), 51−75% (3 points), and 76−100% (4 points). The IRS score was calculated as the intensity score × the distribution score (giving a result from 0 to 12). Cases were divided into two groups for statistical analysis: low ALYREF expression [IRS 0−8] versus high ALYREF expression [IRS 9-12].

### qRT-PCR

Total RNA was extracted from NPC clinical specimens and cell lines using TRIzol reagent. A reverse transcriptase kit (Roche) was used to synthesize cDNA from 1 µg of total RNA. qRT-PCR was conducted using a SYBR Premix ExTaq kit (Takara) on a Light Cycler 480 system (Roche Diagnostics). The relative mRNA levels were calculated using ACTB or GAPDH as the endogenous control to normalize the data. The primer sequences for qRT-PCR are presented in Table S2.

### Western blot analysis

Total protein was dissolved in a sodium dodecyl sulfate sample buffer containing an EDTA-free protease inhibitor cocktail. The secondary antibodies (anti-mouse or anti-rabbit; 1:3000; Thermo) were coupled with horseradish peroxide at 25 °C for 60 min. The target protein bands were detected using enhanced chemiluminescence (ECL) reagent (Millipore) and captured on XAR film. The antibodies used are listed below in the format of name (application, catalog, supplier): anti-ALYREF (WB, HPA019799, Sigma); anti-NOTCH1 (WB, ab52627, Abcam); anti-Flag (WB,14793 S, Cell Signaling Technology); anti-HES1 (WB, AF7575, Affinity Biosciences); anti-HEY1 (WB, ab154077, Abcam); anti-NSUN2 (WB, 20854-1-AP, Proteintech); anti-NSUN6 (WB, 17240-1-AP, Proteintech); anti-ACTB (WB, 3700S, Cell Signaling Technology); anti-GAPDH (WB, 2118S, Cell Signaling Technology).

### Establishment of stable cell lines

We infected cells with a lentivirus vector purchased from GeneCopoeia (Rockville, Maryland, USA) to establish ALYREF overexpression cell lines. NPC cells were treated with a transduction mix containing 0.5 mg/L polybrene. Stable clones were screened with 2 mg/L puromycin, and the efficiency of ALYREF overexpression was validated by qRT-PCR and western blotting.

### Transfection with small interfering RNA (siRNA)

Effective siRNA oligonucleotides targeting ALYREF, NSUN2, and NSUN6, as well as control siRNA (siNC), were obtained from RiboBio (Guangzhou, China). Transient transfection was performed as described previously [23]. The oligonucleotide sequences were as follows: siNC, siN0000001-1-5; siALYREF-1#: GAGGTGGCATGACTAGAAA; siALYREF-2#: ACGACATCATTAAACTGAA; siNSUN2-1#: GAAGCATCGTGCTGAAGTA; siNSUN2-2#: GGGTTATCCTCACAAATGA; siNSUN2-3#: GCATCATGGTGGTCAACCA. siNSUN6-1#: GAACAAAGGCGGTTAAACT; siNSUN6-2#: GCTGCAGCGATTTGATCCA; siNSUN6-3#: CTTAGTAAGTCATGTACTA.

### Transwell invasion and migration assays

Cell invasion and migration assays were conducted in Transwell chambers with an 8-µm polyethylene terephthalate membrane (24-well inserts, NEST, Cat. No.702001). In the upper chamber, 5×10^4^ (migration assay) or 1 × 10^5^ (invasion assay) cells in 200 µL FBS-free RPMI 1640 were seeded, with or without Matrigel (BioCoat, Cat. No. 354480) coating, respectively. The bottom wells were filled with 700 µL RPMI 1640 containing 10% FBS to stimulate invasion or migration. After incubation for 22−24 h, NPC cells at the bottom of the chamber were fixed with methanol for 15 min and stained with 0.5% crystal violet. Then, five fields of vision were selected and photographed with an inverted microscope. To determine the effect of NOTCH1 on the role of ALYREF in NPC metastasis, 50 mg/L of the NOTCH1 inhibitor LY3039478 (Selleckchem Chemicals, USA) was used to treat the HONE1 cells with ALYREF overexpression or vector control cells for 24 h.

### Cell proliferation assay

For the cell proliferation assay, one thousand NPC cells/well were seeded in a 96-well plate, and cell viability was detected using a Cell Counting Kit-8 (CCK-8) (Sigma, St Louis, MO, USA) according to the manufacturer’s instructions. Cell proliferation curves were plotted based on the absorbance of each well at 450 nanometers every 24 h for 5 days.

### RNA BS-Sanger sequencing

Total RNA from NPC cells was isolated using Trizol reagent. RNA bisulfite treatment assays were performed using the EZ RNA Methylation kit (R5002, Zymo Research). The bisulfite-treated RNA and untreated control RNA were reverse-transcribed using SuperScript II RT (18064-071, Invitrogen). Methylation-specific PCR was carried out to identify the m5C modification sites of NOTCH1 mRNA. The cDNA reverse-transcribed from the bisulfite-treated RNA and untreated control RNA was used for PCR with primer pairs indicated in Table S2. The detailed PCR conditions were described in a previous study [24]. The PCR products were purified and ligated into the pEASY-T1 simple cloning vector. After transforming Escherichia coli, the cloned PCR products were subjected to Sanger sequencing.

### RNA immunoprecipitation (RIP)

The RIP assay was performed using the Magna RIP^TM^ RNA-binding protein Immunoprecipitation Kit. The Flag-RIP in HONE1 cells with ALYREF overexpression and vector control cells were respectively cultured in 10-cm dishes for 24 h. The cells were then lysed, and the lysate was incubated with anti-FLAG beads at 4 °C overnight.

For Flag-RIP in the ALYREF overexpressing and control HONE1 cells with NSUN2 or NSUN6 knockdown, the cells were cultured in 10-cm dishes for 12 h, then transfected with siNSUN2, siNSUN6, and siNC, respectively. After 48 h of transfection, the cells were lysed, and the lysate was incubated overnight with anti-FLAG beads at 4 °C.

For the Flag-RIP of wild-type or mutant NOTCH1 mRNA, 293 T cells were co-transfected with the designated plasmids for 48 h. Then, the cells were lysed and incubated with anti-Flag beads (A8592, sigma) at 4 °C overnight. Afterward, the beads were washed five times, and the RNA was collected by adding TRIzol reagent. Finally, qRT–PCR was performed on the input sample (1%) and all the RIP RNA samples. The relative enrichment was determined by calculating the 2^−ΔCt^ of the RIP sample relative to the input sample. The plasmids of WT-NOTCH1 and MUT-NOTCH1 were constructed by inserting the wild-type sequence or the mutant sequence into the PGL3-control plasmid, respectively. The corresponding primers of wild-type sequence and the mutant sequence were shown in Table S3.

### m5C-RIP

HONE1 cells were plated into 10-cm dishes and cultured for 12 h. Then, the cells were transfected with siRNAs (siNSUN2 and siNC) for 48 h, after which the total RNA was isolated using TRIzol reagent. For m5C-RIP, the total RNA was incubated with an anti-m5C antibody (ab10805, Abcam) or mouse IgG antibody (ab131368, Abcam) coupled with Protein A/G Dynabeads in 1 ml of RIP buffer (50 mM Tris–HCl, 750 mM NaCl and 0.5% NP-40) at 4 °C overnight. The beads were washed five times with RIP buffer, and the bound RNA was collected by adding TRIzol reagent. Finally, qRT-PCR was performed using the input sample (1%) and all RIP RNA samples. The relative enrichment was determined by calculating the 2^−ΔCt^ of the RIP sample relative to the input sample.

### Measurement of mRNA stability

HONE1 cells with ALYREF overexpression and control cells were grown in 12-well plates overnight and treated with actinomycin D (2 mg/L, A9415, Sigma). RNA was collected at 0, 0.5, 1.5 and 2.5 h after actinomycin D treatment and analyzed by qRT-PCR. The expression of mRNA in each group was calculated and normalized to GAPDH at the corresponding time.

### Animal experiments

BALB/c-nu mice (4 weeks old) were purchased from Beijing Vital River Laboratory Animal Technology Co., Ltd. (Beijing, China). We used HONE1 cells stably expressing luciferase (HONE1-luci), including HONE1-luci-vector and HONE1-luci-ALYREF. An inoculum comprising 1 × 10^6^ cells in 0.1 ml PBS was subcutaneously injected into the tail vein of nude mice. After 6 weeks, D-luciferin (Potassium Salt D, ThermoFisher) was diluted to 15 mg/ml with PBS, and 100 µL of the resulting solution was injected into each mouse. Subsequently, the IVIS Lumina imaging system (Caliper Life Sciences) was used to assess lung metastasis via bioluminescence imaging (BLI). All animal experiments were carried out in accordance with the regulations approved by the Animal Care and Use Ethnic Committee of SYSUCC (Approval No. L102012021040B).

### Bioinformatics analysis

Total RNA was extracted from SUNE1 and SUNE2 cells transfected with ALYREF-specific siRNA. RNA sequencing (RNA-seq) was carried out by Berry Genomics (Beijing, China). The RNA-seq reads were normalized using the RSEM method [25]. Gene set enrichment analysis (GSEA) was used to predict ALYREF-related pathways and gene sets in NPC. The raw sequence data has been deposited into the Sequence Read Archive (SRA, No. PRJNA1016294). To analyze the relationship between ALYREF and NOTCH1 at the mRNA level, we used publicly available RNA profile data from NPC tissues (HRA000035) [26]. Differential expression analysis of m5C-associated genes in NPC versus non-NPC tissues was performed using public microarray data with the accession numbers GSE12452, GSE53819, and GSE61218 in the GEO database (http://www.ncbi.nlm.nih.gov/geo/).

### Statistical analysis

Student’s *t*-test was used to assess the statistical significance of differences between two groups, and Pearson’s method was used for correlation analysis. The Kaplan−Meier method was used to calculate the survival rate, and the log-rank test was used to test for intergroup differences. The Cox proportional hazards regression model was used to determine independent prognostic factors. The ROC curve was generated using MedCalc software and a two-tailed *t-*test was performed on the data. A *P* < 0.05 was considered to indicate statistically significant differences. Statistical significance was indicated with asterisks: *P < 0.05, **P < 0.01, ***P < 0.001; NS, not significant.

## Results

### ALYREF is upregulated in NPC tissues and associated with poor prognosis in NPC patients

**Fig. 1:**
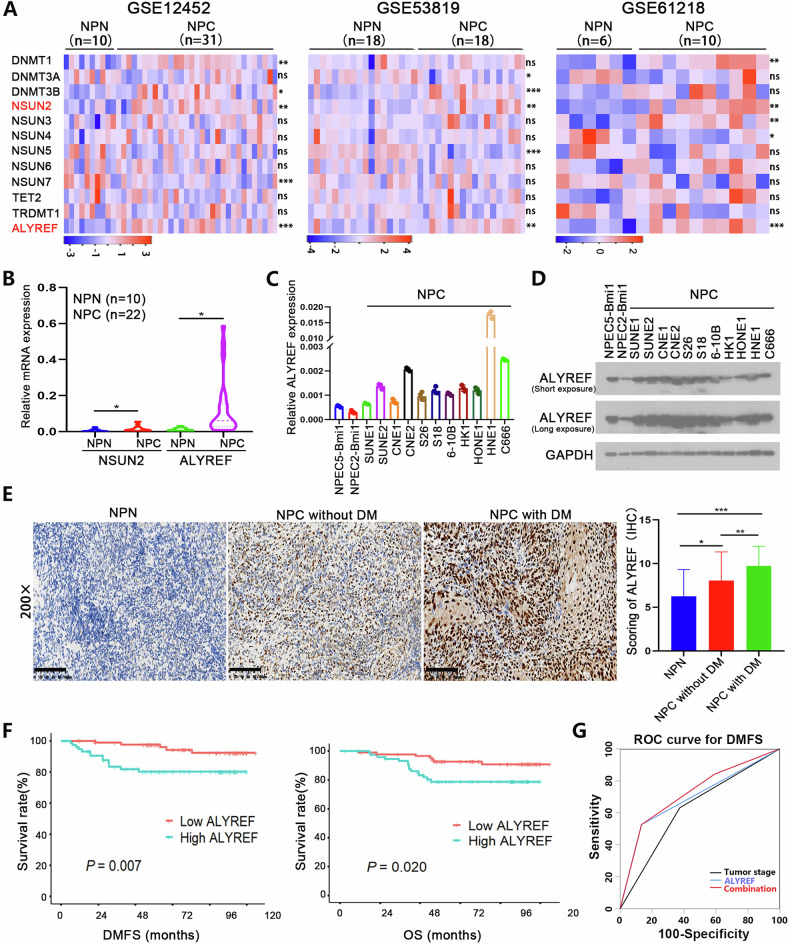
ALYREF is upregulated in NPC tissues and its high expression is associated with a poor prognosis in NPC patients. **A** The expression levels of the m5C-related genes in NPC and NPN tissues from the 3 GEO databases (GSE12452, GSE53819, and GSE61218). **B** The mRNA levels of NSUN2 and ALYREF in NPC (*n* = 22) and NPN tissues (*n* = 10). **C** The mRNA levels of ALYREF in Bmi1-immortalized primary nasopharyngeal epithelial cell lines (NPEC2-Bmi1 and NPEC5-Bmi1) and NPC cell lines. **D** Protein levels of ALYREF in Bmi1-immortalized primary nasopharyngeal epithelial cell lines (NPEC2-Bmi1 and NPEC5-Bmi1) and NPC cell lines. **E** Representative IHC staining results of ALYREF are shown on the left, and the IHC scores of ALYREF are shown on the right. The results are based on 22 normal nasopharyngeal tissues, 161 nonmetastatic NPC tissues, and 31 metastatic NPC tissues. Scale bars represent l00 µm. **F** Kaplan−Meier survival curves of DMFS and OS for 161 NPC patients classified according to the ALYREF expression levels in tumor biopsies. **G** Time-dependent receiver operating characteristic (ROC) analysis showing the clinical risk score (TNM stage), the ALYREF risk score, and the combined clinical and ALYREF risk scores in the NPC cohort. NPC, nasopharyngeal carcinoma; NPN, non-NPC tissues; DMFS, distant metastasis‑free survival; OS, overall survival. DM, distant metastases. *P < 0.05, **P < 0.01, and ***P < 0.001.

To investigate the differential expression of m5C-related genes in NPC, we analyzed three microarray datasets from the GEO database (GSE12452, GSE53819, and GSE61218), comparing NPC and non-NPC tissues. We found that NSUN2 and ALYREF were significantly upregulated in NPC tissues (Fig. 1A). Further analysis of 22 NPC and 10 non-NPC clinical tissue samples revealed that NSUN2 and ALYREF were significantly upregulated in NPC tissues (Fig. 1B), consistent with the results of the GEO dataset analysis. Recently, NSUN2 was found to be correlated with distant metastasis of NPC [27], while the function and regulation of the RNA m5C reader protein ALYREF in NPC were largely unexplored. Thus, we next examined the ALYREF expression levels in 11 NPC cell lines and two non-malignant epithelial cell lines. The results of qRT-PCR (Fig. 1C) and western blot analysis (Fig. 1D) confirmed that ALYREF was upregulated in the NPC cell lines. These results suggest that ALYREF may play an oncogenic role in NPC progression.

We then analyzed ALYREF protein levels in nasopharyngeal tissue samples from 192 NPC patients (161 patients without metastasis, 31 with distant metastasis) and 22 non-NPC people. The expression of ALYREF in NPC tissues was significantly higher than in non-NPC tissues, with the highest expression observed in the nasopharyngeal tissues from patients with distant metastasis (Fig. 1E). Based on the IHC results, the 161 non-metastatic NPC patients were divided into two subgroups according to the median score of ALYREF (using a score of 8 as the cut-off point), categorizing them as high or low ALYREF. Survival analysis suggested that patients with high ALYREF expression had shorter distant metastasis-free survival (DMFS) and overall survival (OS) (Fig. 1F). Further analysis indicated that high ALYREF expression was an independent adverse prognostic factor for DMFS (HR, 3.19; 95% CI, 1.14–8.92; *P* = 0.027) and OS (HR, 2.79; 95% CI, 1.07–5.42; *P* = 0.047) in NPC patients. Moreover, the combination of ALYREF and tumor staging provided additional predictive value for DMFS in NPC patients compared with tumor staging alone (Fig. 1G). A chi-squared test revealed that the rate of high ALYREF expression in the metastatic group was significantly higher than in the non-metastatic group (Table S4). In summary, our results suggest that the upregulation of ALYREF is significantly associated with distant metastasis and poor prognosis in NPC.

### ALYREF promotes NPC metastasis

**Fig. 2:**
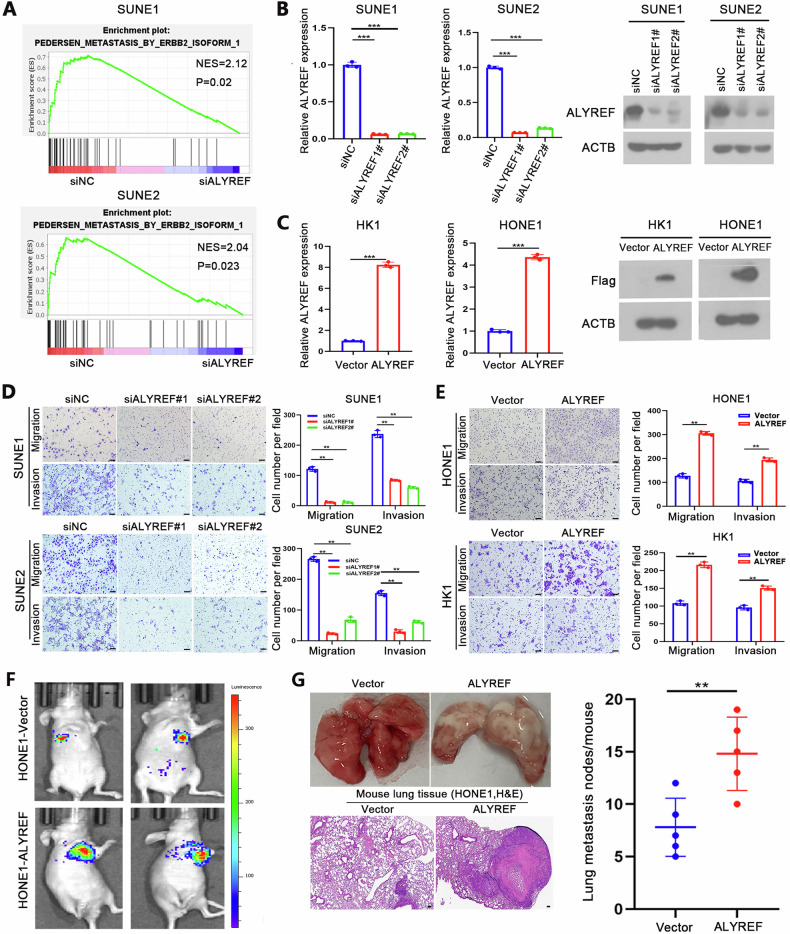
ALYREF promotes NPC cell invasion and metastasis. **A** GSEA results of our RNA-Seq analysis revealing that ALYREF expression is positively correlated with metastases. **B** Knockdown efficiency of ALYREF in SUNE1 and SUNE2 cells was confirmed by qRT-PCR (left panel) and western blotting (right panel). **C** Overexpression efficiency of ALYREF in HONE1 and HK1 cells was assessed by qRT-PCR (left panel) and western blotting (right panel). **D** The results of transwell migration and invasion assays in SUNE1 and SUNE2 cells with or without ALYREF knockdown. Representative data from three independent biological replicates are shown in the left panel. Scale bar: 100 μm. **E** The results of transwell migration and invasion assays in HONE1 and HK1 cells stably overexpressing ALYREF or vector control. Representative data from three independent biological replicates are shown in the left panel. Scale bar: 100 μm. **F** Representative images of a lung metastasis xenograft model using HONE1 cells with ALYREF overexpression or vector control in nude mice in vivo. **G** The number of metastatic nodules in the lungs of mice injected with HONE1 cells stably overexpressing ALYREF or vector control (*n* = 5 in each group). Representative images and quantification of macroscopic tumor nodules on the surface of lung tissues and in lungs stained with H&E in the left panel. Scale bars, l00 µm. *P < 0.05, **P < 0.01 and ***P < 0.001.

To explore the function of ALYREF in NPC, we used siRNA to knock down ALYREF in SUNE1 and SUNE2 cells, after which we performed RNA-seq analysis. The GSEA results indicated that ALYREF knockdown inhibited the expression of genes associated with tumor metastasis (Fig. 2A). To confirm these results, we knocked down ALYREF in SUNE1 and SUNE2 cells (Fig. 2B), as well as overexpressed ALYREF in HK1 and HONE1 cells (Fig. 2C). The results of transwell assays showed that ALYREF knockdown significantly inhibited the invasion and migration abilities of SUNE1 and SUNE2 cells (Fig. 2D). Conversely, overexpression of ALYREF in HK1 and HONE1 cells increased their invasion and migration abilities (Fig. 2E). Additionally, we conducted CCK-8 assays to detect the effect of ALYREF knockdown and overexpression on NPC cell proliferation. Our data showed that cell viability of the ALYREF knockdown and overexpression cells was not significantly altered compared with the corresponding control cells (Fig. S1), suggesting that the promotion of NPC metastasis by ALYREF is not due to an effect on cell proliferation. Furthermore, we used a nude mouse xenograft model of lung metastasis to explore the function of ALYREF in vivo. We observed stronger luciferase signals (Fig. 2F) and more metastatic lung nodules (Fig. 2G) in the ALYREF overexpressed group than in the vector control group, indicating that ALYREF overexpression promoted the lung metastasis of HONE1 cells. Collectively, these results revealed a crucial role of ALYREF in facilitating NPC metastasis.

### ALYREF exerts its oncogenic effect by activating the NOTCH1 signaling pathway

**Fig. 3:**
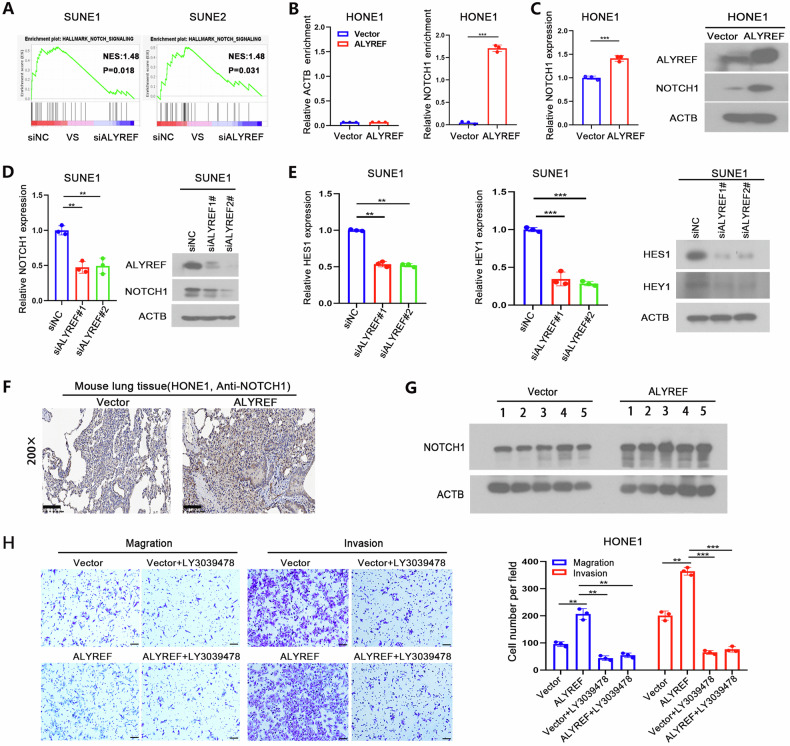
ALYREF promotes NPC metastasis by activating NOTCH1. **A** GSEA results of SUNE1 and SUNE2 cells with ALYREF knockdown (siALYREF) or siRNA control (siNC). **B** The relative Flag-RIP enrichment ratio of NOTCH1 mRNA in HONE1 cells with ALYREF overexpression or vector control. ACTB was used as the negative control. Fold enrichment was determined by calculating the 2^-ΔCt^ of each RIP sample relative to the corresponding input sample. The mRNA and protein levels of NOTCH1 in NPC cells with ALYREF overexpression (**C**) and ALYREF knockdown (**D**) were determined using qRT-PCR and western blotting, respectively. **E** The mRNA and protein levels of downstream targets (HES1, HEY1) of the Notch signaling pathway in SUNE1 cells with ALYREF knockdown or the siNC control were determined by qRT-PCR and western blotting, respectively. **F** Representative immunohistochemical images of lung tissues isolated from mice injected with vector control HONE1 cells (left) or ALYREF-overexpressing cells (right) via the tail vein, confirming the high expression of NOTCH1 in the ALYREF overexpression group. **G** The protein levels of NOTCH1 in lung metastatic nodules with ALYREF overexpression or vector control in nude mice (*n* = 5 per group) were determined by western blot analysis. **H** Inhibition of the NOTCH1 pathway abrogated the ALYREF-mediated upregulation of NPC cell invasion and metastasis. HONE1-overexpressing and corresponding vector control cells were treated with 50 mg/L LY3039478 for 24 h. Scale bar: 100 μm. *P < 0.05, **P < 0.01 and ***P < 0.001.

To investigate the molecular mechanism through which ALYREF promotes NPC metastasis, we performed GSEA analysis on our RNA-Seq data, which revealed a positive correlation between ALYREF and the Notch signaling pathway (Fig. 3A). Since NOTCH1 was previously found to enhance NPC metastasis [28], we hypothesized that ALYREF might exert its role by activating the Notch signaling pathway. We then performed a Flag-RIP assay in HONE1 cells with ALYREF overexpression as well as vector control cells. Compared with the known m5C-free ACTB control mRNA [29], ALYREF protein pulldown yielded a 37-fold enrichment of NOTCH1 mRNA (Fig. 3B). Notably, the overexpression of ALYREF increased the protein and mRNA levels of NOTCH1 in HONE1 cells (Fig. 3C). Conversely, ALYREF knockdown inhibited the protein and mRNA expression of NOTCH1 in SUNE1 cells (Fig. 3D). Furthermore, downstream targets of the Notch signaling pathway, such as HES1 and HEY1, were substantially inhibited in SUNE1 cells upon ALYREF knockdown as shown by qRT-PCR and western blot analysis (Fig. 3E). We validated the presence of metastatic lung xenografts by histological staining and confirmed an increased level of NOTCH1 protein in tumor-bearing mice with ALYREF overexpression compared to control conditions (Fig. 3F, G). To determine whether NOTCH1 inhibition could reverse the ALYREF phenotype, we treated the HONE1 cells with ALYREF overexpression and control with 50 mg/L of LY3039478, a novel r-secretase inhibitor used to block the NOTCH1 signaling pathway. Our results showed that LY3039478 treatment significantly abrogated the enhancement of invasion and migration in ALYREF-overexpressing HONE1 cells (Fig. 3H). In summary, these results indicate that ALYREF promoted NPC metastasis by activating the Notch signaling pathway.

### ALYREF stabilized NOTCH1 mRNA in an m5C-dependent manner

**Fig. 4:**
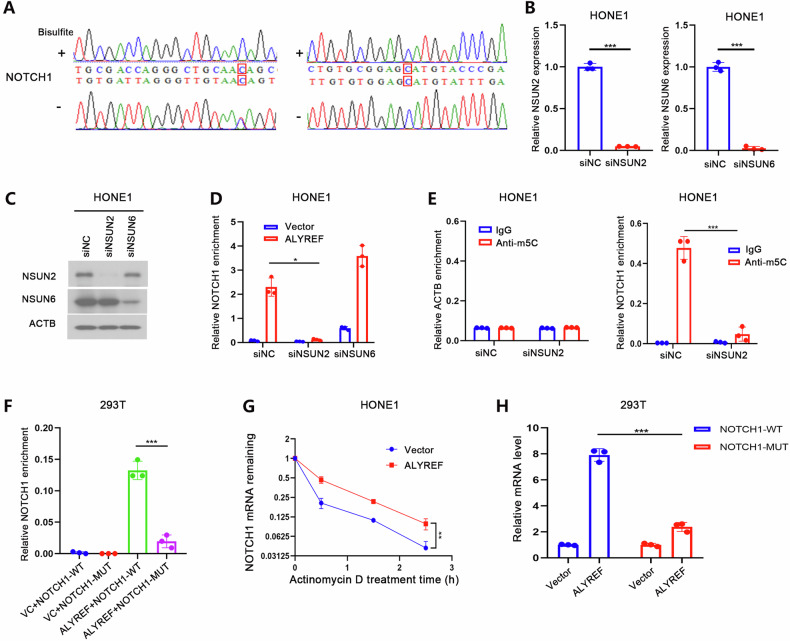
ALYREF stabilizes NOTCH1 mRNA in an m5C-dependent manner. **A** The m5C sites within NOTCH1 (at position chr9:13939412 and 13939452) were identified via RNA BS-Sanger sequencing. The cDNA was amplified by PCR using conventional primers for untreated mRNA and specific primers for bisulfite-treated mRNA. Knockdown efficiency of NSUN2 and NSUN6 in HONE1 cells was determined by qRT-PCR (**B**) and western blot analysis (**C**). **D** The relative Flag-RIP enrichment ratio of the NOTCH1 mRNA in ALYREF-overexpressing and vector control HONE1 cells transfected with siNSUN2, siNSUN6, or siNC, respectively. The RIP assays were performed using an anti-Flag antibody after 48 h of siRNA transfection. Fold enrichment was determined by calculating the 2^−ΔCt^ of the RIP sample relative to the corresponding input sample. **E** The relative IgG or anti-m5C antibody enrichment of the NOTCH1 mRNA in HONE1 cells transfected with siNSUN2 or siNC siRNA. The RIP assays were performed using an antibody specific for m5C or IgG control. ACTB was used as the negative control. Fold enrichment was determined by calculating the 2^−ΔCt^ of the RIP sample relative to the corresponding input sample. **F** The relative Flag-RIP enrichment of the NOTCH1 mRNA was measured in 293 T cells co-transfected with plasmids encoding wild-type or mutant NOTCH1 combined with the plasmid encoding ALYREF or vector control for 24 h. The fold Flag-RIP enrichment was determined by calculating the 2^−ΔCt^ of the RIP sample relative to the corresponding input sample. **G** Residual mRNA levels of NOTCH1 after termination of transcription via actinomycin D treatment in ALYREF overexpressed or vector control HONE1 cells. **H** 293 T cells were co-transfected the ALYREF plasmid and the wild-type or mutant NOTCH1 plasmids for 24 h. The relative mRNA levels were normalized to the housekeeping gene GAPDH. Experiments were independently repeated three times, and the results are presented as the means ± SD of *n* = 3 biological replicates. *P < 0.05, **P < 0.01 and ***P < 0.001.

As an m5C reader, the biological function of ALYREF depends on the m5C modification of target mRNAs. Our previous findings indicated that ALYREF could bind to NOTCH1 mRNA and regulate its expression. Therefore, we assumed that NOTCH1 mRNA might contain m5C modification sites. In this study, two m5C modification sites of NOTCH1 mRNA (at positions chr9: 13939412 and 13939452) were identified using an RNA BS-Sanger sequencing assay (Fig. 4A). As NSUN2 and NSUN6 were previously well established as m5C methyltransferases of mRNA, we used siRNA libraries (mix of 3 different siRNA) to knock down their expression in ALYREF-overexpressing HONE1 cells (Fig. 4B, C), followed by a Flag-RIP assay. The results indicated that the enrichment ratio of ALYREF on NOTCH1 mRNA was significantly reduced by NSUN2 knockdown, whereas no such effect was observed for NSUN6 knockdown (Fig. 4D). Similarly, NSUN2 knockdown significantly reduced the enrichment ratio of anti-m5C antibodies on NOTCH1 mRNA (Fig. 4E). To further confirm the m5C modification sites on NOTCH1 mRNA, plasmids encoding site-specifically mutated or wild-type NOTCH1 mRNA were constructed by cloning equal-length fragments containing the two potential m5C sites or mutant fragments with C → G mutations at both potential sites. The results of the Flag-RIP assay indicated that ALYREF protein pulldown contained more wild-type NOTCH1 mRNA than the mutant control (Fig. 4F). To monitor the effects of ALYREF on the half-life of the NOTCH1 mRNA, ALYREF-overexpressing and control HONE1 cells were treated with the RNA synthesis inhibitor actinomycin D. The results of qRT-PCR analysis suggested that ALYREF overexpression increased the stability of NOTCH1 mRNA (Fig. 4G). To further determine whether the regulation of NOTCH1 mRNA expression by ALYREF required m5C modification, we co-transfected 293 T cells with the ALYREF plasmid and the wild-type or mutant NOTCH1 plasmids, respectively. We found that ALYREF significantly increased the expression level of wild-type NOTCH1 transcripts compared to the mutant control transcripts (Fig. 4H), suggesting that the promotion of NOTCH1 expression by ALYREF might be dependent on the m5C modification and direct ALYREF binding. Collectively, our results indicate that ALYREF interacts with NOTCH1 mRNA and increases its stability in an m5C-dependent manner.

### Clinical relevance of NSUN2, NOTCH1 and ALYREF in NPC

**Fig. 5:**
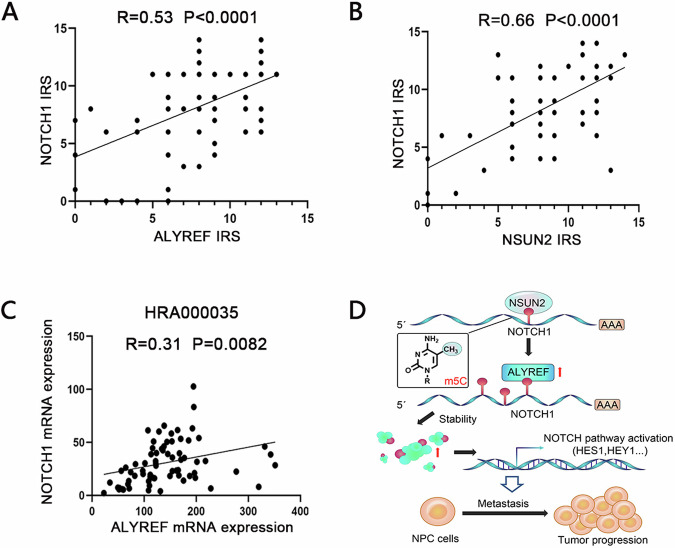
Clinical correlation between NSUN2, NOTCH1 and ALYREF in NPC. **A** The correlation between NOTCH1 and NSUN2, as well as (**B**) NSUN2 and ALYREF protein levels was assessed using 92 paraffin-embedded human NPC tissues. **C** The correlation between NOTCH1 and ALYREF expression at the mRNA level in NPC tissues was assessed using a publicly available RNA profile data (HRA000035). **D** Working model of the proposed mechanism based on the results of this study. In NPC cells, NSUN2 mediates the m5C modification of NOTCH1 mRNA, after which ALYREF cats as an m5C reader protein that increases its stability. Finally, the increased NOTCH1 protein levels promote NPC cell metastasis through the activation of the Notch signaling pathway, leading to NPC progression. Notably, the oncogenic function of ALYREF could be abrogated using the NOTCH1 inhibitor LY3039478. *P < 0.05, **P < 0.01, ***P < 0.001.

To determine the relationship between ALYREF and NOTCH1 expression levels in NPC tissues, we performed IHC assays to measure the corresponding protein levels in FFPE samples from NPC. The IHC analysis showed a high correlation of NOTCH1 with both ALYREF (Fig. 5A) and NSUN2 protein levels (Fig. 5B). In addition, we analyzed the relationship between ALYREF and NOTCH1 at the mRNA level using publicly available RNA profile data (HRA000035) of NPC tissues and found that the mRNA expression levels of ALYREF were positively correlated with NOTCH1 expression levels in NPC tissues (Fig. 5C). These data strongly suggest that m5C modification regulates NOTCH1 in NPC tissues.

Based on our data, we propose a working model of RNA m5C modification in NPC cells, as illustrated in Fig. 5D. In this model, NSUN2 mediates m5C modification of NOTCH1 mRNA. Subsequently, ALYREF acts as a reader protein that recognizes the m5C-modified NOTCH1 mRNAs to maintain their stability. Finally, the increased NOTCH1 protein levels promote metastasis by activating the Notch signaling pathway. Based on these findings, ALYREF may serve as a useful biomarker for NPC prognosis and may guide the use of the NOTCH1 inhibitor LY3039478 in clinical practice.

## Discussion

NPC is a primary nasopharyngeal tumor prone to metastasis, with a particularly high incidence in southern China, Southeast Asia, and North Africa. Distant metastasis is one of the main causes of death in NPC patients. Therefore, it is urgent to identify the key molecular alterations leading to distant metastasis in NPC and provide effective therapeutic targets. Previous studies have confirmed that regulatory factors affecting m5C methylation are key players in various disease processes [30]. In this study, we found that NSUN2 and ALYREF were significantly upregulated in NPC tissues, suggesting their potential oncogenic role in NPC progression. A recent study revealed that NSUN2 expression levels were correlated with distant metastasis of NPC, while also demonstrating that NSUN2 promoted the proliferation, migration, and invasion of NPC cells in vitro [27]. However, the function and regulation of ALYREF in NPC remained largely unexplored, which inspired the conception of this study.

The ALYREF gene, also known as THOC4, is located on 17q25.3 and encodes a ubiquitous nuclear chaperone protein that controls many biological processes. Previous studies demonstrated that ALYREF expression is dysregulated in various tumors [14, 31], while inhibition of ALYREF expression could lead to decreased cell proliferation [14] and migration in oral squamous carcinoma cells [15]. Our results revealed that ALYREF was significantly upregulated in NPC, whereby its high expression was correlated with poor OS and DMFS of NPC patients. In addition, the RNA-seq data analysis that ALYREF was related to metastasis. Functional studies confirmed that ALYREF promoted the metastasis of NPC cells in vitro and in vivo. Consequently, our results indicate that ALYREF plays an important role in NPC metastasis, making it a potential unfavorable prognostic factor and therapeutic target for NPC.

Dysregulation of epigenetic modification mechanisms, including RNA modifications, DNA methylation, nucleosome positioning, as well as histone variants and modifications [32], has been associated with the occurrence and development of cancers. The m5C modification is one of the most common post-transcriptional modifications in human mRNA and non-coding RNA [33]. Recently, RNA m5C modification has increasingly been recognized as an important regulator of physiological processes such as mRNA stability [7, 8], translation efficiency [9] and nuclear export [10]. Conversely, dysregulation of m5C RNA modification is closely related to various important pathological processes, including cancer progression and development [30]. However, little is known about the effect of m5C modification on NPC. As a distinct m5C reader protein, ALYREF has been shown to promote the stability and storage of its target RNAs in a m5C-dependent manner [34]. In the current study, we found that the NOTCH1 mRNA was m5C-modified by NSUN2, after which it was recognized and stabilized by ALYREF, thereby promoting NOTCH1 expression and activating the Notch signaling pathway in NPC cells. However, the detailed mechanisms through which ALYREF affects the stability of NOTCH1 mRNA are not clear and warrant further investigation in the future.

Notably, NOTCH1 is the most frequently detected member of the NOTCH receptor family in tumor tissues and is the most widely studied [35]. Previous studies have found that NOTCH1 is a key driving factor for the progression of NPC, while knockdown of NOTCH1 inhibited the growth and invasion of NPC cells [19]. However, it is not yet clear how NOTCH1 expression is regulated in NPC. Here, we revealed that NOTCH1 is a direct target of ALYREF in NPC. In addition, we found that treatment with the NOTCH1 inhibitor LY3039478 abrogated the oncogenic function of ALYREF in NPC cells. These findings suggest that LY3039478 holds promise for NPC patients exhibiting increased ALYREF expression.

Taken together, our results identified ALYREF as a novel oncogene in NPC. Upregulation of ALYREF was found to be associated with metastasis and unfavorable prognosis for NPC patients. In NPC cells, ALYREF was found to promote metastasis by activating NOTCH1 in an m5C-dependent manner. Importantly, our findings highlight ALYREF as a potential biomarker and therapeutic target for the treatment of NPC using LY3039478.

### Supplementary information

Supplemental Material

## Data Availability

Key raw data were uploaded onto the Research Data Deposit public platform (RDD), with the approval RDD number of RDDB2024151421. The experimental data presented in the study are included in the article/Supplementary Materials, further inquiries can be directed to the corresponding authors upon reasonable request.
