# Supplementary material for: ALYREF promotes the metastasis of nasopharyngeal carcinoma by increasing the stability of NOTCH1 mRNA
Source: Cell Death Dis. 2024 Aug 8;15(8):578. doi: 10.1038/s41419-024-06959-1 (PMC11310353; doi:10.1038/s41419-024-06959-1)

Supplementary material

Table S1. Baseline patient characteristics for the entire group (n= 161).

Characteristic		No. (%) of patients by ALYREF		*P* value
		Low ALYREF (n = 86)	High ALYREF (n = 75)	
Age, year				0.771
≤ 45	78 (48.4)	43 (50.0)	35 (46.7)	
> 45	83 (51.6)	43 (50.0)	40 (53.3)	
Sex				0.855
Male	121 (75.2)	64 (74.4)	57 (76.0)	
Female	40 (24.8)	22 (25.6)	18 (24.0)	
Smoking				0.411
No	103 (64.0)	58 (67.4)	45 (60.0)	
Yes	58 (36.0)	28 (32.6)	30 (40.0)	
Drinking				0.999
No	142 (88.2)	76 (88.4)	66 (88.0)	
Yes	19 (11.8)	10 (11.6)	9 (12.0)	
Family history of cancer				0.235
No	118 (73.3)	67 (77.9)	51 (68.0)	
Yes	43 (26.7)	19 (22.1)	24 (32.0)	
EBV DNA, copy/mL				0.174
≤ 4000	83 (51.5)	49 (57.0)	34 (45.3)	
> 4000	78 (48.5)	37 (43.0)	41 (54.7)	
Neoadjuvant chemotherapy				0.366
No	86 (53.42)	49 (56.98)	37 (49.33)	
Yes	75 (46.58)	37 (43.02)	38 (50.67)	
Concurrent chemotherapy				0.444

No	30 (18.6)	14 (16.3)	16 (21.3)	
Yes	131 (81.4)	72 (83.7)	59 (78.7)	
T stage				0.339
T1	21 (13.0)	11 (12.8)	10 (13.3)	
T2	27 (16.8)	16 (18.6)	11 (14.7)	
T3	78 (48.5)	45 (52.3)	33 (44.0)	
T4	35 (21.7)	14 (16.3)	21 (28.0)	
N stage				0.413
N0	17 (10.6)	6 (7.0)	11 (14.7)	
N1	83 (51.5)	48 (55.8)	35 (46.7)	
N2	37 (23.0)	19 (22.1)	18 (24.0)	
N3	24 (14.9)	13 (15.1)	11 (14.7)	
Overall stage				0.546
I	7 (4.4)	3 (3.5)	4 (5.3)	
II	26 (16.1)	15 (17.4)	11 (14.7)	
III	74 (46.0)	43 (50.0)	31 (41.3)	
IVA	54 (33.5)	25 (29.1)	29 (38.7)	

Abbreviation:EBV, Epstein-Barr virus

[a]*P* values were calculated using the chi-square test or Fisher exact test if indicated.

[b]According to the 8th edition of the Union for International Cancer Control staging system.

Table S2. Primer sequences used in this study.

**Gene**	**Primer sequences (5'-3')**	**Usage**
ALYREF	Forward, ACATTCAGCTTGTCACGTCAC	qRT-PCR
	Reverse, TCTAGTCATGCCACCTCTGTTTA	
ACTB	Forward, GTGAAGGTGACAGCAGTCGGT	qRT-PCR
	Reverse, AAGTGGGGTGGCTTTTAGGA	
HES1	Forward, TCAACACGACACCGGATAAAC	qRT-PCR
	Reverse, GCCGCGAGCTATCTTTCTTCA	
HES2	Forward, CCAACTGCTCGAAGCTAGAGA	qRT-PCR
	Reverse, AGCGCACGGTCATTTCCAG	
HEY1	Forward, GTTCGGCTCTAGGTTCCATGT	qRT-PCR
	Reverse, CGTCGGCGCTTCTCAATTATTC	
NOTCH1	Forward, TGGACCAGATTGGGGAGTTC	RIP-qPCR
	Reverse, GCACACTCGTCTGTGTTGAC	
NOTCH1	Forward, ACGCAGTCTCTGCAGTGC	BS PCR
	Reverse, CAAGCACCCCATCAAGC	
NOTCH1	Forward, GAAGTACTTCAGTGACGGCCA	BS PCR
	Reverse, CATCAAGCGTGCCGC	
NOTCH1 (bisulfite-treated)	Forward, TGTGTGGAAGGTTAGTGTAATTTTT	BS PCR
	Reverse, CACAACTCCTCCTCACAACCA	
NOTCH1	Forward, TAGTGTGTGGAAGGTTAGTGTAAT	BS PCR

(bisulfite-treated)	Reverse, ACAACTCCTCCTCACAACCAT	

Abbreviations: ALYREF, Aly/REF export factor; NOTCH1, pyruvate kinase muscle isozyme M2; NSUN2, NOP2/Sun RNA methyltransferase family member 2.

Table S3. The corresponding primers of wild-type sequence and the mutant sequence in construction of plasmids.

**Gene**	**Sequence 5'~3'**
WT-NOTCH1	**C**ACGCAGTCTCTGCAGTGCTGGAAGTACTTCAGTGACGGCCACTGTGACAGC<br>CAGTGCAACTCAGCCGGCTGCCTCTTCGACGGCTTTGACTGCCAGCGTGCGGA<br>AGGCCAGTGCAACCCCCTGTACGACCAGTACTGCAAGGACCACTTCAGCGAC<br>GGGCACTGCGACCAGGGCTGCAACAGCGCGGAGTGCGAGTGGGACGGGCTG<br>GACTGTGCGGAGCATGTACCCGAGAGGCTGGCGGCCGGCACGCTGGTGGTGG<br>TGGTGCTGATGCCGCCGGAGCAGCTGCGCAACAGCTCCTTCCACTTCCTGCGG<br>GAGCTCAGCCGCGTGCTGCACACCAACGTGGTCTTCAAGCGTGACGCACACG<br>GCCAGCAGATGATCTTCCCCTACTACGGCCGCGAGGAGGAGCTGCGCAAGCA<br>CCCCATCAAGCGTGCCGCCGAGGGC
MUT-NOTCH1	**C**ACGCAGTCTCTGCAGTGCTGGAAGTACTTCAGTGACGGCCACTGTGACAGC<br>CAGTGCAACTCAGCCGGCTGCCTCTTCGACGGCTTTGACTGCCAGCGTGCGGA<br>AGGCCAGTGCAACCCCCTGTACGACCAGTACTGCAAGGACCACTTCAGCGAC<br>GGGCACTGCGACCAGGGCTGCAAGAGCGCGGAGTGCGAGTGGGACGGGCTG<br>GAGTGTGCGGAGCATGTACCCGAGAGGCTGGCGGCCGGCACGCTGGTGGTGG<br>TGGTGCTGATGCCGCCGGAGCAGCTGCGCAACAGCTCCTTCCACTTCCTGCGG<br>GAGCTCAGCCGCGTGCTGCACACCAACGTGGTCTTCAAGCGTGACGCACACG<br>GCCAGCAGATGATCTTCCCCTACTACGGCCGCGAGGAGGAGCTGCGCAAGCA<br>CCCCATCAAGCGTGCCGCCGAGGGC

Table S4. The relationship between ALYREF expression and metastasis.

Group	Patients (n=192)	ALYREF expression Low-expression (n=95)	High-expression (n=97)	*P-value*
Non-metastatic NPC	161 (84.0%)	86 (90.5%)	75 (77.3%)	0.018
Metastatic NPC	31 (16.0%)	9 (9.5%)	22 (22.7%)	

Fig. S1. Knockdown and overexpressing of ALYREF in NPC cells has no effect on growth in NPC cells *in vitro*. **(A).** Cell growth rates between ALYREF knockdown and control cells were compared by CCK-8 assay. **(B).** Cell growth rates between ALYREF overexpressing and control cells were compared by CCK-8 assay.

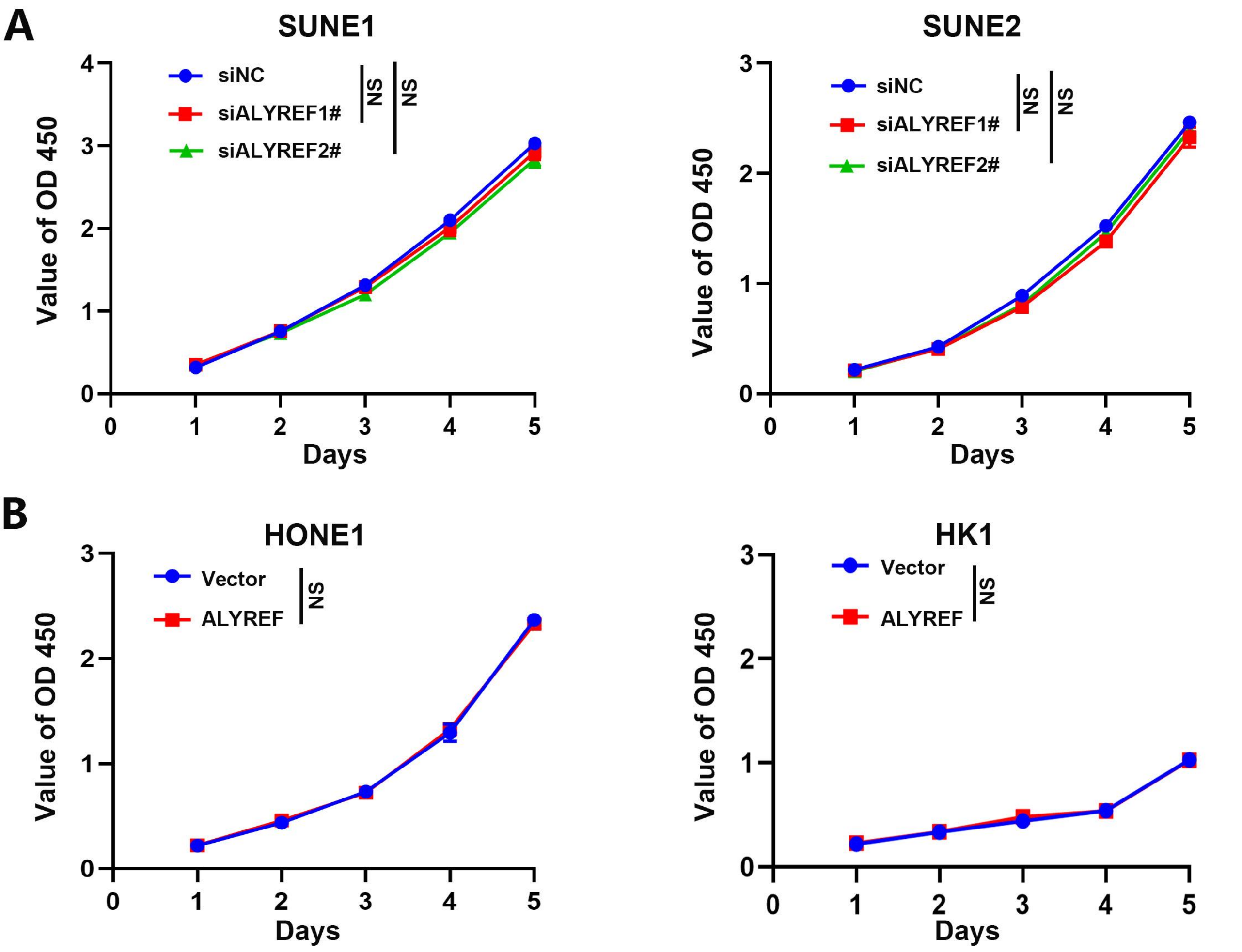

Full length western blots

Fig 1D

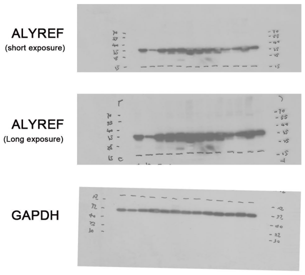

Fig 2B

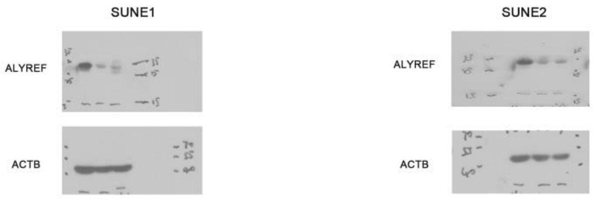

Fig 2C

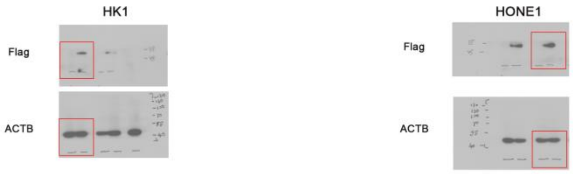

Fig 3C

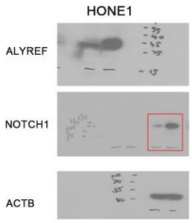

Fig 3D

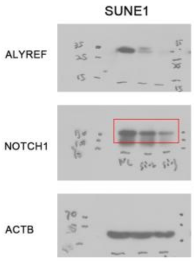

Fig 3E

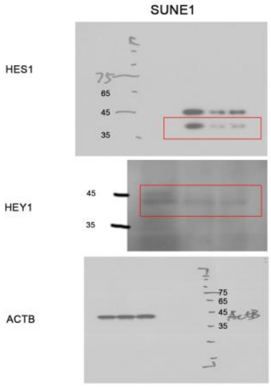

Fig 3G

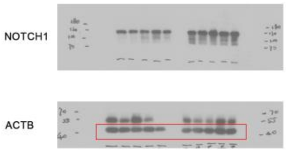

Fig 4C

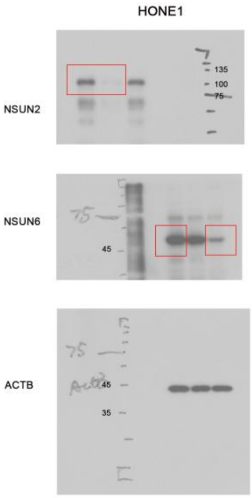

Supplement: Supplementary file 1 — Supplemental Material [file 41419_2024_6959_MOESM1_ESM.pdf]
